# PER1 prevents excessive innate immune response during endotoxin-induced liver injury through regulation of macrophage recruitment in mice

**DOI:** 10.1038/cddis.2016.9

**Published:** 2016-04-07

**Authors:** T Wang, Z Wang, P Yang, L Xia, M Zhou, S Wang, Jie Du, J Zhang

**Affiliations:** 1 Center for Molecular Metabolism, Nanjing University of Science and Technology, Nanjing, China; 2 Cambridge Suda Genome Resource Center, Soochow University, Suzhou, China; 3 Department of Radiology, Affiliated Hospital of Nanjing University of Chinese Medicine, Nanjing, China; 4 Beijing An Zhen Hospital, Capital Medical University, Beijing, China

## Abstract

The severity of acute liver failure (ALF) induced by bacterial lipopolysaccharide (LPS) is associated with the hepatic innate immune response. The core circadian molecular clock modulates the innate immune response by controlling rhythmic pathogen recognition by the innate immune system and daily variations in cytokine gene expression. However, the molecular link between circadian genes and the innate immune system has remained unclear. Here, we showed that mice lacking the clock gene *Per1* (Period1) are more susceptible to LPS/d-galactosamine (LPS/GalN)-induced macrophage-dependent ALF compared with wild-type (WT) mice. *Per1* deletion caused a remarkable increase in the number of Kupffer cells (KCs) in the liver, resulting in an elevation of the levels of pro-inflammatory cytokines after LPS treatment. Loss of *Per1* had no effect on the proliferation or apoptosis of macrophages; however, it enhanced the recruitment of macrophages, which was associated with an increase in CC chemokine receptor 2 (*Ccr2*) expression levels in monocytes/macrophages. Deletion of *Ccr2* rescued d-GalN/LPS-induced liver injury in *Per1*^−/−^ mice. We demonstrated that the upregulation of *Ccr2* expression by *Per1* deletion could be reversed by the synthetic peroxisome proliferator-activated receptor gamma (PPAR-*γ*) antagonist GW9662. Further analysis indicated that PER1 binds to PPAR-*γ* on the *Ccr2* promoter and enhanced the inhibitory effect of PPAR-*γ* on *Ccr2* expression. These results reveal that *Per1* reduces hepatic macrophage recruitment through interaction with PPAR-*γ* and prevents an excessive innate immune response in endotoxin-induced liver injury.

Acute liver failure (ALF) is characterized by severe hepatic injury with failure of hepatocyte function, resulting in a clinical syndrome of coagulopathy, encephalopathy and circulatory dysfunction. ALF is associated with high overall mortality, ranging from 30 to 80%.^1^ Bacterial lipopolysaccharide (LPS) is implicated in the pathogenesis of ALF. LPS enters the liver through the portal blood flow and promotes the hepatic innate immune response. As key components of the hepatic innate immune system, Kupffer cells (KCs) are postulated to have a central role in response to LPS. Upon stimulation by LPS, KCs secrete pro-inflammatory cytokines, including interleukin 1 (IL-1), IL-6, monocyte chemoattractant protein 1 (MCP-1) and tumor necrosis factor (TNF)-*α*. Many of these pro-inflammatory mediators can trigger hepatocyte cell death and lead to ALF.^2, 3^

Recent studies in mice demonstrated that the actions of macrophages in ALF largely depend on the recruitment of monocytes and macrophages into the liver.^4, 5^ Chemokines may be critically involved in this process of leukocyte recruitment and activation. Leukocytes sense concentration gradients and move toward increasing chemokine concentrations. One of the most potent chemokines identified for monocytes and macrophages is MCP-1, which acts by binding to the CC chemokine receptor 2 (CCR2). Evidence from both murine and human studies suggests that CCR2/MCP-1 chemotaxis is responsible for the mobilization and subsequent trafficking of a population of activated monocytes/macrophages to the liver from the bone marrow.^6, 7, 8^

Circadian rhythms are reflected by daily oscillations of multiple biological processes including the immune response. The fundamental mechanism of rhythm generation is highly conserved. Interlocked transcriptional/translational feedback loops involving clock genes, such as *Per1–3*, *Cry1–2*, *Clock*, *Bmal1* and *Rev-Erbα*, create oscillations on the molecular level.^9, 10^ In mice, significant temporal dependence of LPS-induced endotoxic shock has been reported.^11^ The nuclear receptor *Rev-Erbα* mediates selective circadian regulation of inflammatory cytokines.^12^ Innate immune pathogen recognition mechanisms are also under circadian control. The circadian clock controls Toll-like receptor 9-mediated innate and adaptive immunity.^13^ Blood leukocyte numbers have long been known to exhibit circadian oscillations.^14, 15^ Recent studies have revealed that gene expression in macrophages exhibits robust circadian oscillation.^16^

Given the intimate association between the innate immune response and circadian rhythms, we explored the role of the clock gene *Per1* (Period1) in ALF induced by administration of d-galactosamine (GalN)/LPS, which is a well-established model similar to ALF in the clinical setting. The results presented here showed that *Per1*^−/−^ mice develop more severe d-GalN/LPS-induced inflammatory liver damage, as evidenced by increased production of pro-inflammatory cytokines, as well as more severe liver pathology. The hepatic recruitment of macrophages was enhanced in *Per1*^−/−^ mice, which leads to increased susceptibility to d-GalN/LPS. We further demonstrated that deletion of *Per1* alleviates the inhibitory effect of peroxisome proliferator-activated receptor gamma (PPAR-*γ*) on *Ccr2* expression, resulting in an increase in the number of KCs in *Per1*^−/−^ mice.

## Results

### Loss of *Per1* leads to an increase in d-GalN/LPS-induced lethality

**Figure 1:**
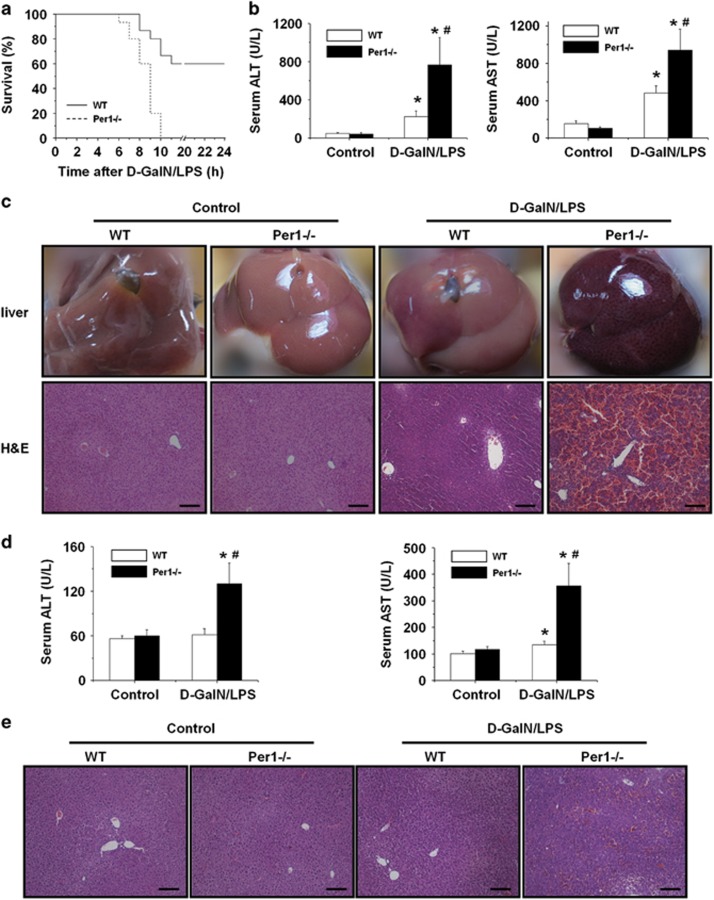
*Per1* protects mice from d-GalN/LPS-induced liver injury and prolongs survival. WT and *Per1*^−/−^ mice were administered 5 *μ*g/kg body weight LPS intraperitoneally in combination with 500 mg/kg body weight d-GalN or PBS as the control. (**a**) Survival was monitored for 24 h (*n*=15 for each group). (**b**) Serum activities of ALT and AST were measured 5 h after d-GalN/LPS challenge. (**c**) Macroscopic appearance of representative liver samples and H&E staining of the different groups (as indicated) at 5 h after d-GalN/LPS challenge. WT mice and *Per1*^−/−^ mice were administered 3 *μ*g/kg body weight LPS intraperitoneally in combination with 200 mg/kg body weight d-GalN or PBS as the control. (**d**) Serum activities of ALT and AST were measured 5 h after d-GalN/LPS challenge. (**e**) H&E staining of representative liver samples is shown. Experiments were repeated independently at least three times with consistent results. In each independent repeat, *n*≥5 (*n*, number of samples in each group). *P<0.05, d-GalN/LPS group *versus* control group; ^#^*P*<0.05, *Per1*^−/−^ group *versus* WT group. Scale bar, 200 *μ*m

To examine the effects of *Per1* loss on the inflammatory response, mice were injected intraperitoneally with LPS in combination with d-GalN. In the *Per1*^−/−^ mice group, mortality became apparent at 5–6 h, and all mice died by 10 h. In the wild-type (WT) mice group, no death was observed at 6 h; the first animal death was observed at 8 h, and the survival rate was 60% at 24 h (Figure 1a). Based on the survival rate, additional WT and *Per1*^−/−^ mice were killed 5 h after d-GalN/LPS administration to obtain blood samples and liver tissues for liver enzyme and tissue analyses. Serum alanine transaminase (ALT) and aspartate transaminase (AST) activities were found to be significantly higher in *Per1*^−/−^ mice than that in WT mice (Figure 1b). Histological examination of the liver tissues revealed more prominent liver damage in *Per1*^−/−^ mice (Figure 1c). In the *Per1*^−/−^ mice group, massive hemorrhagic necrosis and hepatocyte apoptosis were observed, with prominent vascular congestion and inflammatory cell infiltration. In contrast, liver damage and histological changes were found to be significantly less severe in WT mice. Then, we explored the impact of *Per1* on non-lethal liver inflammation induced by d-GalN/LPS treatment. The results showed that none of the WT mice treated with 3 *μ*g/kg LPS and 200 mg/kg d-GalN died. Administration of d-GalN/LPS at this lower dosage caused no apparent liver injury in WT mice. In *Per1*^−/−^ mice, the same dose of d-GalN/LPS induced significant liver injury, as detected by increased transaminase activities and histological changes (Figures 1d and e).

### Loss of *Per1* increases d-GalN/LPS-induced production of inflammatory cytokines and chemokines

**Figure 2:**
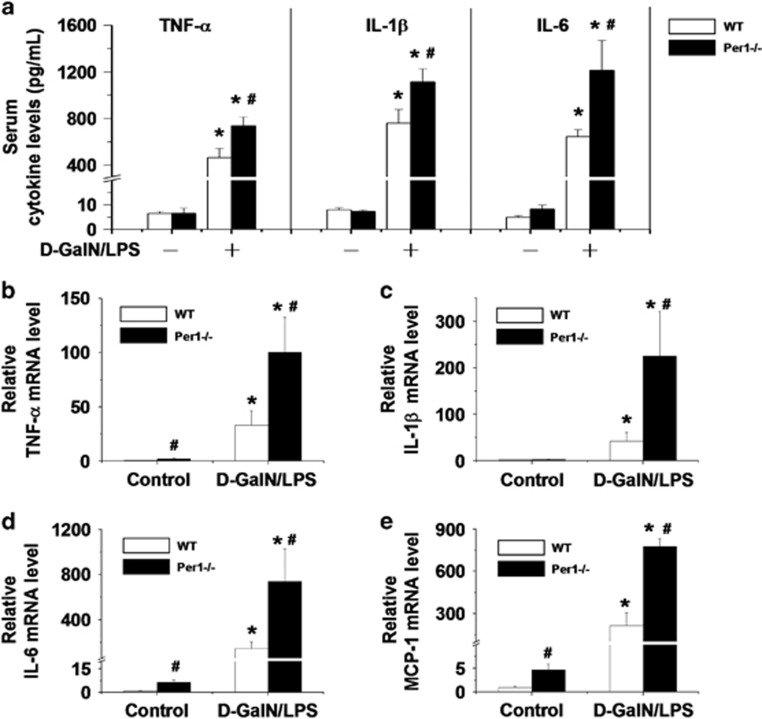
*Per1* deficiency increases the expression of pro-inflammatory cytokines in the liver. Sera and livers of both WT and *Per1*^−/−^ mice were harvested 5 h after i.p. injection of PBS or 5 *μ*g/kg LPS and 500 mg/kg d-GalN. (**a**) Serum TNF-*α*, IL-1*β* and IL-6 were measured by ELISA. (**b**-**e**) The hepatic mRNA levels of TNF-*α*, IL-1*β*, IL-6 and MCP-1 were measured by quantitative RT-PCR. Experiments were repeated independently at least three times with consistent results. In each independent repeat, *n*=5; *P<0.05, d-GalN/LPS group *versus* control group; ^#^*P*<0.05, *Per1*^−/−^ group *versus* WT group

Current models of d-GalN/LPS have associated outcomes with elevated production of inflammatory cytokines; thus, we measured the levels of serum cytokines in mice after d-GalN/LPS administration. Serum TNF-*α*, IL-1*β* and IL-6 were significantly higher in *Per1*^−/−^ mice than in WT mice (Figure 2a). Real-time reverse transcriptase (RT)-PCR analysis of TNF-*α*, IL-1*β*, IL-6 and MCP-1 (Figures 2b–e) revealed that the expression of all these cytokines was markedly elevated in the *Per1*^−/−^ mice either with or without d-GalN/LPS treatment.

### Loss of *Per1* increases the number of KCs in the liver

**Figure 3:**
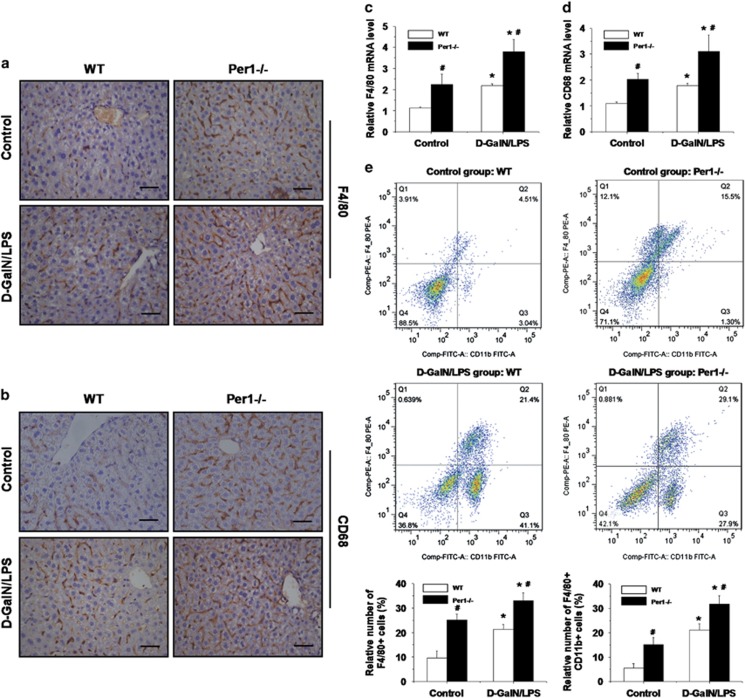
Increased number of macrophages in *Per1*-deficient livers. Liver tissues were harvested from WT and *Per1*^−/−^ mice either under baseline conditions or at 3 h after d-GalN/LPS challenge. Representative immunohistochemical staining of livers was performed using antibodies against the specific macrophage markers F4/80 (**a**) and CD68 (**b**). Scale bar, 50 *μ*m. The hepatic mRNA levels of F4/80 (**c**) and CD68 (**d**) were measured by quantitative RT-PCR. (**e**) Flow cytometric analysis of surface F4/80 and CD11b was used to determine the relative number of macrophages in the livers of mice. *n*=5; *P<0.05, d-GalN/LPS group *versus* control group; ^#^*P*<0.05, *Per1*^−/−^ group *versus* WT group

We then examined the response of *Per1*^−/−^ cells to LPS. Peritoneal macrophages were isolated from WT and *Per1*^−/−^ mice and stimulated with LPS (1 *μ*g/ml). The expression of cytokines in macrophages was measured by real-time RT-PCR at 3 h after stimulation. Unexpectedly, *Per1* deletion had no influence on the expression of any of the cytokines (Supplementary Figure S1). To confirm the phenotypes observed here, RAW264.7 cells were transfected with a plasmid expressing *Per1* by electroporation as described previously.^17^ However, no changes in LPS-induced cytokine production were observed in either of the groups (Supplementary Figure S1). We next determined the number of KCs in the livers of *Per1*^−/−^ and WT mice. Administration of d-GalN/LPS significantly increased the number of KCs in both genotypes. Either under baseline conditions or after d-GalN/LPS challenge, a marked increase was observed in the number of KCs in the livers of *Per1*^−/−^ mice compared with the livers of WT mice, as shown by immunohistochemistry using a specific antibody against the KC marker genes F4/80 and CD68 (Figures 3a and b), as well as by the increased hepatic expression of F4/80 and CD68 (Figures 3c and d). We further used flow cytometry to identify specific populations of myeloid cells in the mouse liver. The data showed a significant increase in total F4/80^+^ cells in untreated and treated *Per1*^−/−^ mice (Figure 3e). F4/80^+^ CD11b^+^ cells showed a strong capacity for the production of cytokines in response to LPS.^18^ The relative number of these cells also increased in *Per1*^−/−^ mice, either under baseline conditions or after d-GalN/LPS treatment (Figure 3e). These results implied that the increase in the number of KCs in *Per1*^−/−^ livers may contribute to the immune response to LPS and to increased hepatic cytokine production.

### *Per1* had no influence on the proliferation or apoptosis of macrophages

The increased number of macrophages in *Per1*-deficient livers could be the result of enhanced proliferation and/or impaired apoptosis of macrophages. Local production of M-CSF in the liver has a crucial role in the proliferation and maturation of KCs.^19^ We found that *Per1* deficiency did not significantly change the hepatic expression of M-CSF (Supplementary Figure S2A). A cell cycle analysis of peritoneal macrophages isolated from WT and *Per1*^−/−^ mice revealed similar numbers of macrophages undergoing the G0/G1, S and G2/M phases of the cell cycle (Supplementary Figure S2). An annexin V/PI assay was performed on macrophages harvested at 2, 4, 7 and 12 days of culture under routine conditions to assess the percentage of early apoptotic (annexin V+/PI−) and late apoptotic/necrotic (annexin V+/PI+) cells. The WT and *Per1*-deficient macrophages displayed similar changes over time (Supplementary Figure S2). Therefore, we concluded that *Per1* has no influence on the proliferation or apoptosis of macrophages.

### *Per1* deficiency increases hepatic *Ccr2* expression and enhances hepatic macrophage migration

**Figure 4:**
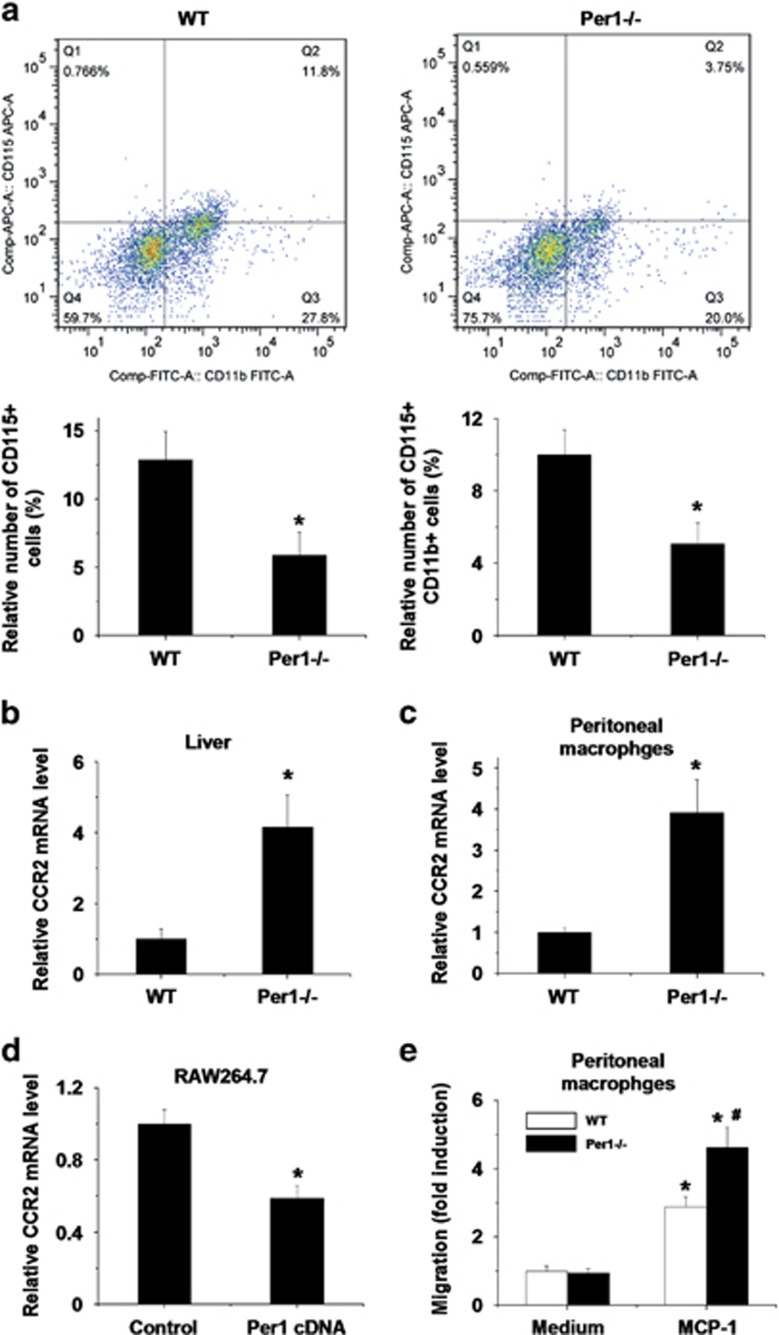
*Per1* inhibits *Ccr2* expression and macrophage migration. (**a**) Flow cytometry analysis of surface CD115 and CD11b was used to determine the relative numbers of monocytes in peripheral blood. *Ccr2* expression was measured in the livers (**b**) and peritoneal macrophages (**c**). *P<0.05, *Per1*^−/−^ group *versus* WT group. (**d**) RAW264.7 cells were transfected with the pCMV-Sport2 vector as the control or pCMV-Sport2 *Per1*, and the mRNA levels of *Ccr2* were measured. *P<0.05, *Per1* cDNA group *versus* control group. (**e**) Peritoneal macrophages isolated from WT or *Per1*^−/−^ mice were placed in the upper chamber. Serum-free media containing MCP-1 (50 ng/ml) was placed in the lower chamber. Migration of the peritoneal macrophages into the lower chamber was determined 12 h after stimulation. *P<0.05, MCP-1 group *versus* medium group; ^#^*P*<0.05, *Per1*^−/−^ group *versus* WT group. Experiments were repeated independently at least three times with consistent results. In each independent repeat, *n*=5

The increased number of KCs could also be due to enhanced monocyte/macrophage recruitment to the liver. FACS analysis revealed a decrease in total CD115^+^ circulating monocytes in the peripheral blood of *Per1*^−/−^ mice. The relative number of CD115^+^ CD11b^+^ monocytes was also decreased in *Per1*^−/−^ mice (Figure 4a). This observation implies that hepatic macrophage recruitment is enhanced in *Per1*^−/−^ mice. It was suggested previously that CCR2/MCP-1 chemotaxis is responsible for the mobilization and subsequent trafficking of monocytes/macrophages to the liver.^6, 7, 8^ We have shown that hepatic MCP-1 expression is significantly upregulated in *Per1*^−/−^ mice (Figure 2e). Similar MCP-1 mRNA levels between WT and *Per1*-deficient peritoneal macrophages suggest that the elevated hepatic MCP-1 expression in *Per1*^−/−^ mice is due to the increased number of macrophages in the liver (Supplementary Figure S1D). Hepatic levels of *Ccr2* were also significantly elevated in *Per1*^−/−^ mice (Figure 4b). *Per1* deficiency increased the gene expression of *Ccr2* in peritoneal macrophages (Figure 4c), and *Ccr2* expression was markedly lower in RAW264.7 cells transfected with *Per1* (Figure 4d). Next, a cell chemotaxis assay was performed on the peritoneal macrophages isolated from WT and *Per1*^−/−^ mice. We found that macrophage chemotaxis was significantly induced by MCP-1 stimulation. Macrophages lacking *Per1* exhibited higher chemotactic activity than WT macrophages (Figure 4e).

### Deletion of *Ccr2* rescues d-GalN/LPS-induced liver injury in *Per1*^−/−^ mice by reducing hepatic macrophage recruitment

**Figure 5:**
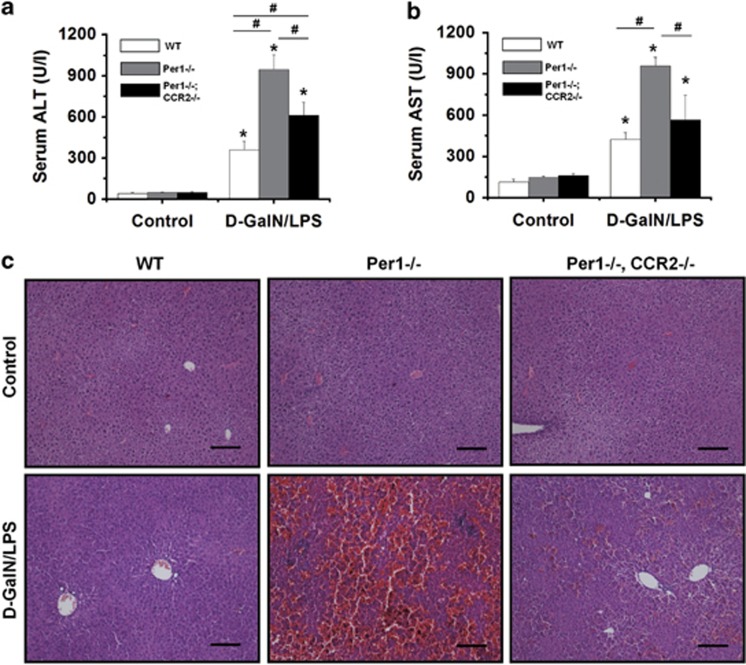
Deletion of *Ccr2* rescues d-GalN/LPS-induced liver injury in *Per1*^−/−^ mice. *Per1*^−/−^ and *Per1*^−/−^; *Ccr2*^−/−^ mice were administered 5 *μ*g/kg LPS and 500 mg/kg d-GalN intraperitoneally; PBS was administered as the control. Serum activities of ALT (**a**) and AST (**b**) were measured at 5 h after d-GalN/LPS challenge. (**c**) H&E staining of representative liver samples is shown. Scale bar, 200 *μ*m. Experiments were repeated independently at least three times with consistent results. In each independent repeat, *n*=5. *P<0.05, significant differences between control group and D-GalN/LPS group; ^#^*P*<0.05, significant differences between genotypes

**Figure 6:**
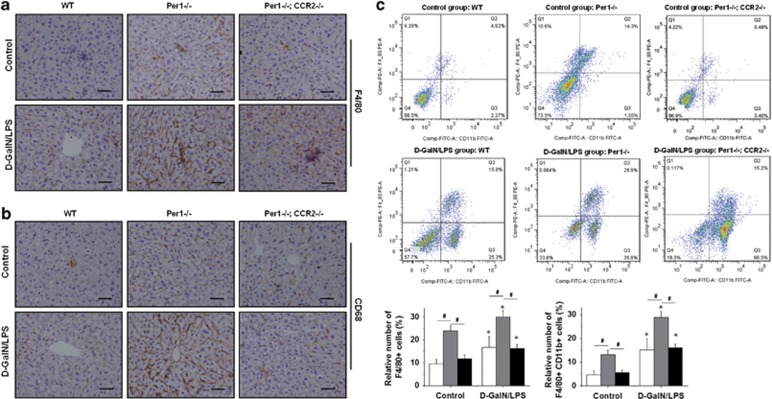
Deletion of *Ccr2* reduces the number of macrophages in *Per1*-deficient livers. Liver tissues were harvested from *Per1*^−/−^ and *Per1*^−/−^; *Ccr2*^−/−^ mice either under baseline conditions or at 3 h after d-GalN/LPS challenge. Representative immunohistochemical staining of livers was performed using antibodies against F4/80 (**a**) and CD68 (**b**). Scale bar, 50 *μ*m. (**c**) Flow cytometry analysis of surface F4/80 and CD11b was used to determine the relative number of macrophages in the livers of mice. *n*=5; *P<0.05, significant differences between control group and D-GalN/LPS group; ^#^*P*<0.05, significant differences between genotypes

To further confirm the association between upregulation of *Ccr2* and elevated susceptibility to d-GalN/LPS in *Per1*^−/−^ mice, we generated *Per1*^−/−^ *Ccr2*^−/−^ (DKO) mice according to Mendel's law. WT, *Per1*^−/−^ and DKO mice were injected with 5 *μ*g/kg LPS and 500 mg/kg d-GalN. Deletion of *Ccr2* significantly rescued d-GalN/LPS-induced liver injury in *Per1*^−/−^ mice, as detected by reduced levels of serum ALT and AST in WT and DKO mice compared with *Per1*^−/−^ mice at 5 h after treatment (Figures 5a and b). Histological examinations of liver sections showed less severe confluent, hemorrhagic necrosis and hepatocyte apoptosis in WT and DKO mice compared with those in *Per1*^−/−^ mice (Figure 5c). We next determined the number of KCs in mice with different genotypes. Deletion of *Ccr2* rescued the abnormal accumulation of KCs in *Per1*^−/−^ mice, as determined by immunohistochemistry for F4/80 and CD68 (Figures 6a and b). Flow cytometry analysis revealed a decrease in total hepatic F4/80^+^ cells in DKO mice, either under baseline conditions or after d-GalN/LPS treatment. A relative decrease in the number of F4/80^+^ CD11b^+^ cells was also observed in DKO mice compared with *Per1*^−/−^ mice. Although the proportions of both hepatic nonparenchymal subsets in DKO mice are similar to those in WT mice (Figure 6c). These results indicated that deletion of *Ccr2* rescues d-GalN/LPS-induced liver injury in *Per1*^−/−^ mice by reducing hepatic macrophage recruitment.

### *Per1* mediates *Ccr2* expression in macrophages through the PPAR-*γ* pathway

**Figure 7:**
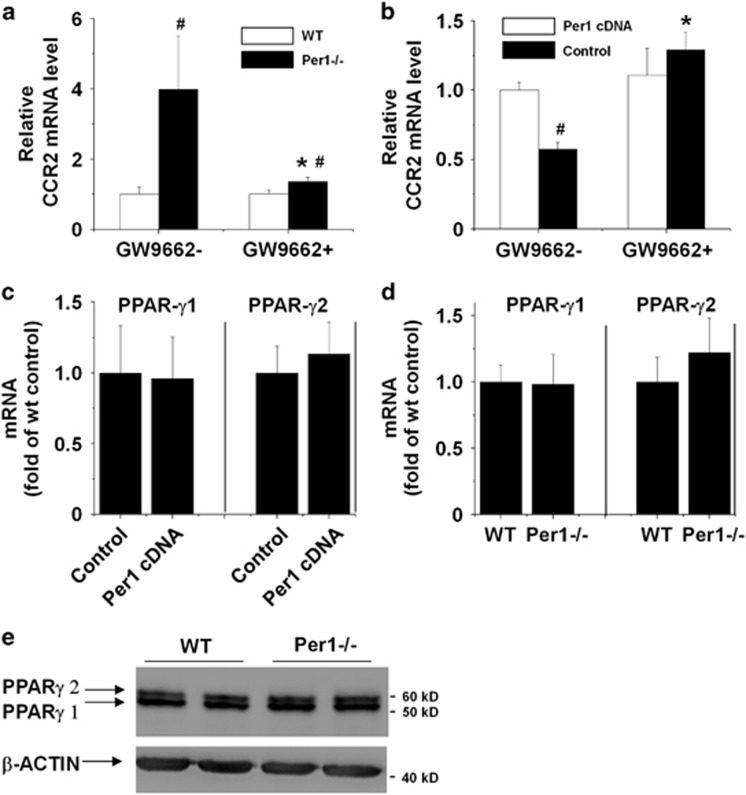
*Per1* inhibits *Ccr2* expression in macrophages through the PPAR-*γ* pathway. After incubating for 3 h with or without 10 *μ*M GW9662, a specific and irreversible PPAR-*γ* inhibitor, the mRNA levels of *Ccr2* in peritoneal macrophages (**a**) and RAW264.7 cells transfected with vector alone or *Per1* cDNA (**b**) were measured. *n*=5; *P<0.05, GW9662+ group *versus* GW9662- group; in **a**, ^#^*P*<0.05, *Per1*^−/−^ group *versus* WT group; in **b**, ^#^*P*<0.05, *Per1* cDNA group *versus* control group. The mRNA levels of PPAR-*γ*1 and PPAR-*γ*2 in peritoneal macrophages (**c**) and RAW264.7 cells transfected with vector alone or *Per1* cDNA (**d**) were measured by quantitative RT-PCR. *n*=5; in **c**, *P<0.05, *Per1*^−/−^ group *versus* WT group; in **d**, *P<0.05, *Per1* cDNA group *versus* control group. (**e**) Western blot analysis was performed on peritoneal macrophages using a PPAR-*γ* antibody, and *β*-actin was used as an internal control

Previous studies reported that *Ccr2* expression was repressed by signaling pathways involving PPAR-*γ* activation.^20, 21, 22^ To investigate whether *Per1* mediates *Ccr2* expression in macrophages through the PPAR-*γ* pathway, macrophages lacking or overexpressing *Per1* were incubated in the presence or absence of 10 *μ*M GW9662, a specific irreversible PPAR-*γ* inhibitor.^20, 21, 22^ We found that GW9662 reversed either the upregulation of *Ccr2* expression by *Per1* deletion in peritoneal macrophages or the downregulation of *Ccr2* expression by *Per1* overexpression in RAW264.7 cells (Figures 7a and b), suggesting that *Per1* regulates *Ccr2* expression in macrophages through the PPAR-*γ* pathway. Real-time RT-PCR and western blot analysis showed that *Per1* had no significant effect on PPAR-*γ* expression in macrophages (Figures 7c–e), implying that *Per1* may mediate *Ccr2* expression by influencing the activation of PPAR-*γ*.

### PER1 interacts with PPAR-*γ*

**Figure 8:**
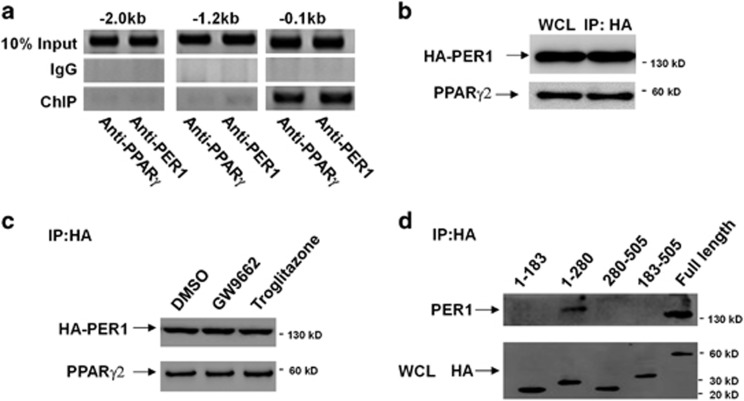
PER1 interacts with PPAR-*γ*. (**a**) Recruitment of PER1 or PPAR-*γ* to the respective target region in the *Ccr2* promoter was detected by a ChIP assay in peritoneal macrophages isolated from WT mice. (**b** and **c**) Vectors expressing HA-tagged PER1 and PPAR-*γ*2 were transfected into B6F10 cells. Immunoblots showed recovery of PPAR-*γ*2 from B6F10 cells after immunoprecipitation with an anti-HA antibody. In **c**, GW9662 (10 *μ*M) or troglitazone (10 *μ*M) was added 3 h before the cells were harvested. (**d**) B6F10 cells were transfected with vectors expressing the corresponding HA-tagged fragments of PPAR-*γ*2 and a vector expressing PER1. Immunoblots showed recovery of PER1 from B6F10 cells after immunoprecipitation with an anti-HA antibody. WCL, whole-cell lysate; IP, immunoprecipitated

A ChIP mapping experiment indicated that both PER1 and PPAR-*γ* bind specifically to only the –180/+16-bp region of *Ccr2* promoter (Figure 8a), implying that PER1 protein may interact with PPAR-*γ*. Vectors expressing HA-tagged PER1 and PPAR-*γ*2 were transfected into B6F10 cells. Immunoblots showed recovery of PPAR-*γ*2 from B6F10 cells after immunoprecipitation with an anti-HA antibody. As expected, we discovered a physical association between PER1 and PPAR-*γ*2 (Figure 8b). Addition of troglitazone (a synthetically specific PPAR-*γ* ligand)^23, 24, 25^ or GW9662 did not significantly alter the association between these two proteins in immunoprecipitation assays (Figure 8c). Finally, we used a series of HA-tagged deletion mutants of PPAR-*γ*2 and investigated their interaction with PER1. Our results showed that amino acids 1–280 of PPAR-*γ*2 interact strongly with PER1, and a further deletion of 97 amino acids (1–183) abolished the ability of PPAR-*γ*2 to bind to PER1 (Figure 8d), demonstrating that residues 183–280 of PPAR-*γ*2 directly interact with PER1. However, residues 183–505, which also contain amino acids 183–280, could not bind to PER1. Despite not directly binding to PER1, residues 1–183 of PPAR-*γ*2 may be indispensable to proper structure of PPAR-*γ*2 and its association with PER1.

## Discussion

Clock regulators appear to have an intimate role in the innate immune response aside from their role in circadian control. Published studies have clearly shown that leukocyte recruitment promotes LPS-induced lethality.^26, 27^ Blood leukocyte numbers have long been known to exhibit circadian oscillations.^14, 15^ Recent studies indicated that circadian rhythms modulate the innate immune system by regulating leukocyte recruitment.^26^ In this study, our results revealed that *Per1* prevents widespread overactivation of the LPS-induced innate immune response by reducing excessive hepatic macrophage recruitment. Loss of *Per1* significantly increased d-GalN/LPS-induced liver damage, which is essentially caused by high levels of pro-inflammatory cytokines, and resulted in elevation of mouse lethality. We demonstrated that the elevated cytokine production in *Per1*^−/−^ mice is due to an increased number of macrophages in the liver. *Per1* deletion resulted in excessive hepatic macrophage recruitment by upregulating *Ccr2* expression through the PPAR-*γ* pathway.

Earlier studies have demonstrated that the secretion of TNF-*α* and its binding to TNFR-I are essential for both lethality and hepatic injury in LPS-induced hepatitis.^28^ Higher levels of LPS-induced hepatic TNF-*α* and other pro-inflammatory cytokines, such as IL-1*β* and IL-6, were observed in *Per1*^−/−^ mice compared with WT mice, leading to more prominent liver damage and lethality in *Per1*^−/−^ mice. It has been reported that the nuclear receptor Rev-Erb*α* mediates selective circadian regulation of inflammatory cytokines.^12^ These reports inspired us to investigate whether *Per1* directly regulates the expression of pro-inflammatory cytokines in the innate immune response to LPS. However, *in vitro* experiments revealed that *Per1* has no effect on the expression of pro-inflammatory cytokines in mice macrophages, suggesting the higher hepatic levels of cytokines in *Per1*^−/−^ mice may be due to the increased number of cells responding to LPS in the liver. Thus, loss of *Per1* markedly increased the number of KCs in mice livers. F4/80^+^ CD11b^+^ cells showed a strong capacity for the production of cytokines in response to LPS.^19^ Flow cytometry analysis also revealed an increase in the number of F4/80^+^ CD11b^+^ cells in *Per1*^−/−^ mice, either at baseline conditions or after d-GalN/LPS administration. This observation also well explained the elevation of hepatic cytokine expression in *Per1*^−/−^ mice without d-GalN/LPS treatment.

Macrophages and their precursors, monocytes, have an important role during inflammatory injury. Monocytes migrate to the inflamed/necrotic area and subsequently differentiate into mature macrophages.^29, 30, 31^ Even in steady-state conditions, KCs are constantly replenished by blood monocytes.^32^ Peripheral blood monocytes are a heterogeneous population of circulating leukocytes. There are two functional subsets among murine blood monocytes: a short-lived CX3CR1^lo^ CCR2^+^ Gr1^+^ subset that is actively recruited to inflamed tissues and a CX3CR1^hi^ CCR2^−^ Gr1^−^ subset characterized by CX3CR1-dependent recruitment to non-inflamed tissues.^33^ In this study, the decline in *Cx3cr1* expression in *Per1*-deficient monocytes/macrophages could not directly contribute to the increased number of KCs in *Per1*^−/−^ mice (data not shown). Previous studies demonstrated that *Ccr2*^−/−^ mice exhibit decreased macrophage infiltration following acetaminophen-induced liver injury,^34^ indicating that MCP-1/CCR2 signaling is essential for monocyte migration to the injured liver. Increasing experimental evidence suggests that the chemokine receptor *Ccr2* regulates monocyte entry into inflamed tissue, mainly indirectly, by promoting the egress of monocytes from the bone marrow into the circulation.^6, 7, 8^ In this study, we demonstrated that the expression of *Ccr2* is significantly inhibited by *Per1*. MCP-1/CCR2 chemotaxis activity was enhanced in *Per1*-deficient macrophages. Deletion of *Ccr2* reduced the number of KCs in *Per1*^−/−^ mice. d-GalN/LPS-induced severe liver damage in *Per1*^−/−^ mice was also ameliorated by *Ccr2* deletion. These results further confirmed that the increased number of KCs in *Per1*^−/−^ mice is due to the elevation of *Ccr2* expression. The increase in the number of KCs in intact *Per1*^−/−^ mice implied that CCR2 may also participate in the replenishment of KCs in *Per1*^−/−^ mice under steady-state conditions. Although it has been reported that *Per1* has an important role in the cell cycle and apoptosis in cancer cells,^26^ the increase in KCs may also be attributed to the influence of *Per1* on proliferation and/or apoptosis in macrophages. Here, we showed that *Per1* alters neither the cell cycle nor apoptosis in macrophages.

*Ccr2* gene expression is regulated by several signaling pathways, including calcineurin/NFAT,^35^ C/EBP*α*^36^ and PPAR-*γ*.^20, 21, 22^ It has been reported that activation of macrophage PPAR-*γ* results in alterations of chemokine receptors such that CCR2 is inactivated and CX3CR1 is activated, resulting in cessation of CCR2-dependent migration and activation of CX3CR1-dependent anchorage to coronary artery smooth muscle cells.^22^ As *Per1* was found to have opposite roles in *Ccr2* and *Cx3cr1* expression (data not shown) in macrophages, we postulated that *Per1* may suppress *Ccr2* expression through the PPAR-*γ* pathway. Our results indicated that the effect of *Per1* on *Ccr2* expression was reversed by the synthetic PPAR-*γ* antagonist GW9662, which strongly supports our hypothesis.

The ChIP assay clearly showed that PER1 protein and PPAR-*γ* bound specifically to only the –180/+16-bp region of the *Ccr2* promoter. In contrast to transcriptional activation, trans-repression does not involve binding to typical receptor-specific response elements; however, PPAR-*γ* and transcription factors bind each other via protein–protein interactions, thus modulating their transcriptional activity.^37, 38^ No potential typical PPAR-γ DNA-binding site was identified in the –180/+16-bp region of the *Ccr2* promoter, indicating that PPAR-*γ* represses the transcription of *Ccr2* by interacting with transcription factors. According to previous studies,^35, 36^ NFAT and C/EBP*α* can promote the transcription of *Ccr2*, and their binding sites in the *Ccr2* promoter overlap with this PPAR-*γ* binding region. Therefore, we speculated that PPAR-*γ* may interact with one of these transcription factors and thus indirectly bind to the *Ccr2* promoter. Further studies are required to support this hypothesis. mPERs have been reported to directly bind to nuclear receptors; however, they do not directly bind to DNA.^39^ PER2 has been reported to interact with the *β*-subunit of casein kinase 2,^40^ glycogen synthase kinase 3*β*,^41^ REV-ERB and possibly other nuclear receptors.^39, 40, 41, 42, 43^ PER3 interacts with PPAR-γ via an N-terminal region including both PAS domains and a preceding predicted helix-loop-helix motif.^44^ The recruitment of PER1 to the PPAR-*γ* binding region in the *Ccr2* promoter implied that PER1 may bind to PPAR-*γ* and stabilize the association between PPAR-*γ* and the transcription factor on the *Ccr2* promoter. In this study, co-immunoprecipitation assays revealed a physical association between PER1 and PPAR-*γ*. The addition of troglitazone or GW9662 did not significantly alter the association between these two proteins, indicating that PER1 binds to PPAR-*γ* in a ligand-independent manner. Further investigation revealed that PPAR-*γ* utilizes part of its DNA-binding and hinge domains (183–280), but not its ligand-binding domain (310–505), to bind PER1. This result further confirmed that the association between PER1 and PPAR-*γ* is ligand independent. Overall, the evidence indicates that PPAR-*γ* has an inhibitory effect on several pro-inflammatory cytokines via interaction with transcription factors such as NF-κB, AP-1 and STAT.^38^ In this article, we showed that *Per1* suppresses *Ccr2* expression through the PPAR-*γ* pathway but has no effect on the expression of cytokines in macrophages, suggesting that *Per1* does not participate in the association between PPAR-*γ* and transcription factors that functionally promote the transcription of these pro-inflammatory cytokines.

In summary, PER1 bound to PPAR-*γ* and stabilized the association between PPAR-*γ* and the transcription factor on the *Ccr2* promoter. Deletion of *Per1* alleviated the inhibitory effect of PPAR-*γ* on *Ccr2* expression, resulting in enhanced macrophage recruitment into the liver. The increased number of KCs caused elevation of cytokine production in d-GalN/LPS-induced liver failure. Thus, the circadian gene *Per1* could protect the liver from inflammatory damage by minimizing the KC-mediated immune response.

## Materials and Methods

### Animals

*Per1*^−/−^ mice^45^ were obtained from Dr. CC Lee at Baylor College of Medicine, Houston, TX, USA. *Ccr2*^−/−^ mice were obtained from Jackson Lab (Bar Harbor, ME, USA). The *Per1*^−/−^; *Ccr2*^−/−^ (DKO) mice were generated at the expected Mendelian ratios and developed normally. All of the animals were backcrossed for at least five generations before the first pilot study to ensure a largely homogenous C57BL/6J background. Male WT C57BL/6J mice and gene knockout mice at 8–10 weeks of age were used in this work. The animals were maintained in cycles of 12 h of light and 12 h of darkness with free access to food and water *ad libitum*. All animal care and use procedures were in accordance with the guidelines of the Institutional Animal Care and Use Committee at Nanjing University of Science and Technology.

### Endotoxin-induced fulminant hepatitis model

Fulminant hepatitis in mice was established by intraperitoneal injection of LPS (5 *μ*g/kg body weight; Sigma-Aldrich, St. Louis, MO, USA) and d-GalN (500 mg/kg body weight; Sigma-Aldrich). To determine the survival rate, the animals were monitored continuously after LPS/d-GalN injection until their death. For assessment of liver damage, the animals were killed 5 h after treatment with this dose to obtain blood and liver tissue.

To induce non-lethal liver inflammation, mice were intraperitoneally injected with LPS (3 *μ*g/kg body weight) and d-GalN (200 mg/kg body weight). Blood and liver samples were collected 5 h after treatment.

### Serum aminotransferase activity and ELISA

Serum AST and ALT activities were measured using an AU2700 automatic biochemical analyzer (Olympus, Tokyo, Japan). Serum TNF-*α*, IL-1*β* and IL-6 levels were determined using enzyme-linked immunosorbent assay (ELISA) kits (Boster Biological Technology Ltd, Wuhan, China) according to the manufacturer's protocol.

### Antibody

For immunohistochemical staining, purified antibodies were obtained from Abcam (Shanghai, China) and eBioscience (San Diego, CA, USA) (anti-mouse CD68, Abcam, cat no. ab955; anti-mouse F4/80, eBioscience, cat no. 14–4801). Antibodies for flow cytometry analysis were obtained from eBioscience and BD Biosciences (San Jose, CA, USA) (anti-mouse F4/80, PE-conjugated, eBioscience, cat no. 12–4801; anti-mouse CD11b, FITC- conjugated, BD Biosciences, cat no. 557396; anti-mouse CD115, APC-conjugated, eBioscience, cat no. 17-1152). Antibodies for western blots and immunoprecipitation assays were obtained from Sigma-Aldrich, Cell Signaling Technology (Shanghai, China), Abcam and Bioworld Technology (Nanjing, China) (anti-HA, Sigma-Aldrich, cat no. H3663; anti-mouse PPAR*γ*, Cell Signaling Technology, cat no. 2430; anti-mouse PER1, Abcam, cat no. ab3443; anti-mouse *β*-actin, Bioworld Technology, cat no. AP0060).

### Histological analysis

Liver tissue was fixed in 10% phosphate-buffered formalin overnight, embedded in paraffin and cut into 4-*μ*m sections. The sections were stained with hematoxylin and eosin (H&E). CD68 and F4/80 were used as immunohistochemical markers for KCs in the liver as described previously.^46^

### Cell culture and treatment

Peritoneal macrophages were isolated from mice by peritoneal lavage 4 days after injection of 2 ml of 3% thioglycolate as described previously.^47^ Peritoneal macrophages and RAW264.7 cells were maintained in RPMI-1640 supplemented with 10% low-endotoxin FBS and stimulated with LPS (1 *μ*g/ml) for the indicated time periods.

### RNA extraction and quantitative real-time PCR

Total RNA was extracted from the samples with Trizol (Invitrogen, Carlsbad, CA, USA) according to the manufacturer's instructions. The reverse transcription reaction was carried out using a reverse transcriptase kit (Invitrogen) according to the manufacturer's protocol. Real-time PCR was performed, and the products were detected using the ABI 7300 Detection System with SYBR Green dye (Toyobo, Osaka, Japan). The expression level of glyceraldehyde-3-phosphate dehydrogenase was simultaneously quantified as an internal standard control. Gene expression in monocytes/macrophages was normalized against B2M and TBP as previously described.^48^ The sequences of all primers used for quantitative RT-PCR are shown in Supplementary Table S1.

### Flow cytometry

For flow cytometry experiments, hepatic inflammatory cells and peripheral blood mononuclear cells (PBMCs) were prepared as described previously.^29^ Hepatic inflammatory cells were stained with fluorescently labeled antibodies against F4/80 and CD11b. PBMCs were incubated with fluorescently labeled antibodies against CD115 and CD11b. Flow cytometric analysis was performed by using a FACScan flow cytometer (BD Biosciences) at Nanjing Medical University.

For cell cycle analysis, cells in the G0/G1, S and G2/M phases of the cell cycle were identified using Vybrant Dye Cycle violet stain (Invitrogen) according to the manufacturer's protocol. The proportion of apoptotic cells was measured using a FACScan flow cytometer according to the instructions provided in the Annexin V/PI kit (Invitrogen).

### Cell chemotaxis assay

The cell chemotaxis assay was performed as previously described.^49^ Peritoneal macrophages were placed in the upper chambers of the transwell (5 × 10^4^ cells per well) and exposed to medium or MCP-1 (50 ng/ml, R&D Systems, Shanghai, China) in the lower chamber. After a 12-h incubation, the number of migrated cells was determined by blinded observers, who counted 10 microscopic fields per well.

### Western blot analysis

Proteins were extracted following the procedure described previously.^50^ The proteins were separated by SDS-PAGE on 8–12% polyacrylamide gels and subsequently electrically transferred to a PVDF membrane. After blocking with 5% (w/v) BSA in TBST at room temperature for 1 h, the membranes were then incubated with an appropriate specific primary antibody (anti-HA, 1 : 2000; anti-PPAR-*γ*, 1 : 1000; anti-PER1, 1 : 200; anti-*β*-actin, 1 : 1000) at 4 °C overnight, followed by incubation with an HRP-conjugated secondary antibody (1 : 10 000). Detection was performed using an enhanced chemiluminescence kit (Thermo Scientific, Hudson, NH, USA).

### Chromatin immunoprecipitation assays

The ChIP assay was performed as described previously with slight modifications.^51^ RAW264.7 cells were plated in 100-mm dishes and stimulated with LPS for 1 h. Cross-linked chromatin was immunoprecipitated with 5 *μ*g of a specific antibody (anti-PPAR-*γ*; anti-PER1) or negative control rabbit IgG (Millipore, Billerica, MA, USA) at 4 °C overnight. Immunoprecipitated DNA was then used as a template for PCR. The sequences of all primers used for ChIP-PCRs are listed in Supplementary Table S2.

### Co-immunoprecipitation

For co-immunoprecipitation, B6F10 cells were transfected with the indicated plasmids using Lipofectamine 2000 Transfection Reagent (Invitrogen) in 10-cm dishes. Co-immunoprecipitation was performed as described previously.^52^ Briefly, cells were lysed with a solution containing 10 mM Tris-HCl (pH 8), 420 mM NaCl, 1 mM EDTA and 0.5% NP-40 with protease inhibitor cocktail (Boster Biological Technology Ltd). To prepare the immunoprecipitates, we incubated the cell lysates with an anti-HA monoclonal antibody (Sigma-Aldrich) overnight at 4 °C followed by incubation with Protein A-Sepharose 4B (Invitrogen). The immunoprecipitates were washed five times with wash buffer containing 10 mM Tris-HCl pH 8, 100 mM NaCl, 1 mM EDTA, 0.5% NP-40 and 0.5% Triton X-100 and subsequently boiled in SDS-PAGE loading buffer. The proteins were analyzed by western blotting as described above.

### Statistical analysis

The statistical significance of lethality was analyzed using the Kaplan–Meier method. Groups of data are presented as mean±S.D. One-way analysis of variance followed by Dunnett's test or Student's *t*-test was used to determine the statistical significance of difference in measured parameters. Difference was considered significant at *P*<0.05.

## Abbreviations

*Per1*: Period1
GalN: galactosamine
LPS: lipopolysaccharide
CCR2: CC chemokine receptor 2
IP: immunoprecipitation
PPAR-*γ*: peroxisome proliferator-activated receptor gamma
ALF: acute liver failure
KCs: Kupffer cells
MCP-1: monocyte chemoattractant protein 1
WT: wild-type
TNF: tumor necrosis factor
IL: interleukin
RT: reverse transcriptase
