# Supplementary material for: PER1 prevents excessive innate immune response during endotoxin-induced liver injury through regulation of macrophage recruitment in mice
Source: Cell Death Dis. 2016 Apr 7;7(4):e2176–. doi: 10.1038/cddis.2016.9 (PMC4855679; doi:10.1038/cddis.2016.9)
Supplement: Supplementary Information [file cddis20169x1.doc]

**Supplementary Figure 1.** The expression of pro-inflammatory cytokine in macrophages. (A-D) Peritoneal macrophages were isolated from both WT and *Per1*-/- mice by peritoneal lavage. Cells were treated with LPS (1 μg/mL). The mRNA levels of TNF-α, IL-1β, IL-6 and MCP-1 were measured by quantitative RT- PCR at 3 h after LPS stimulation. *P<0.05, LPS+ group versus LPS- group; #P<0.05, *Per1*-/- group versus WT group. (E) RAW264.7 cells were transfected with pCMV-Sport2 vector as control or pCMV-Sport2 *Per1* by electroporation. The mRNA levels of TNF-α, IL-1β and IL-6 were measured by quantitative RT- PCR at 3 h after LPS stimulation (1 μg/mL). *P<0.05, LPS+ group versus LPS- group; #P<0.05, *Per1* cDNA group versus control group. All experiments were repeated independently at least three times with consistent results. In each independent repeat, n=5. (F) The transfection efficiency in RAW264.7 cells.

**Supplementary Figure 2.** *Per1* has no influence on the proliferation and apoptosis of macrophages. (A) The hepatic mRNA levels of M-CSF were measured by quantitative RT- PCR (n=5). Peritoneal macrophages were isolated from both WT and *Per1*-/- mice. (B and C) Cells were stained with Vybrant Dye Cycle DNA stain, and cell cycle analysis was performed. (D and E) Cells were harvested 2d, 4d, 7d and 12d after cultured under routine conditions respectively, and the annexinV/PI assay was performed to assess the percentage of early apoptotic (annexinV+/PI-) and late apoptotic/necrotic (annexinV+/PI+) cells. *P<0.05, *Per1*-/- group versus WT group.
