# Supplementary material for: PER1 prevents excessive innate immune response during endotoxin-induced liver injury through regulation of macrophage recruitment in mice
Source: Cell Death Dis. 2016 Apr 7;7(4):e2176–. doi: 10.1038/cddis.2016.9 (PMC4855679; doi:10.1038/cddis.2016.9)
Supplement: Supplementary Tables [file cddis20169x2.doc]

Table S1 Primer sequences used for quantitative RT-PCR

| gene | Forward/ Reverse | Primer (5’ to 3’) |
| --- | --- | --- |
| TNF-α | Forward | ACGGCATGGATCTCAAAGAC |
|  | Reverse | CGGACTCCGCAAAGTCTAAG |
| IL-1β | Forward | CATCCAGCTTCAAATCTCGCAG |
|  | Reverse | CACACACCAGCAGGTTATCATC |
| IL6 | Forward | CATGTTCTCTGGGAAATCGTGG |
|  | Reverse | GTACTCCAGGTAGCTATGGTAC |
| MCP-1 | Forward | CTGGATCGGAACCAAATGAG |
|  | Reverse | AAGGCATCACAGTCCGAGTC |
| F4/80 | Forward | CTTTGGCTATGGGCTTCCAGTC |
|  | Reverse | GCAAGGAGGACAGAGTTTATCGTG |
| CD68 | Forward | CTTCCCACAGGCAGCACAG |
|  | Reverse | AATGATGAGAGGCAGCAAGAGG |
| M-CSF | Forward | CCCATATTGCGACACCGAA |
|  | Reverse | AAGCAGTAACTGAGCAACGGG |
| CX3CL1 | Forward | ACGAAATGCGAAATCATGTGC |
|  | Reverse | CTGTGTCGTCTCCAGGACAA |
| CCR2 | Forward | TCATCCACGGCATACTATCAACA |
|  | Reverse | GTGGCCCCTTCATCAAGCT |
| CX3CR1 | Forward | ttcattggcttctttgggg |
|  | Reverse | atgttgacttccgagttgcg |
| PPAR-γ1 | Forward | GCGGCTGAGAAATCACGTTC |
|  | Reverse | GAATATCAGTGGTTCACCGCTTC |
| PPAR-γ2 | Forward | AACTCTGGGAGATTCTCCTGTTGA |
|  | Reverse | GAAGTGCTCATAGGCAGTGCAT |
| GAPDH | Forward | CATCCACTGGTGCTGCCAAGGCTGT |
|  | Reverse | ACAACCTGGTCCTCAGTGTAGCCCA |
| B2M | Forward | TTCTGGTGCTTGTCTCACTGA |
|  | Reverse | CAGTATGTTCGGCTTCCCATTC |
| TBP | Forward | GAGCTGTTTGCAGACAAAGTTC |
|  | Reverse | CCCTGGCACATGAATCCTGG |

Table S2 Primer sequences used for PCR in ChIP

| CCR2 promoter | Forward/ Reverse | Primer (5’ to 3’) |
| --- | --- | --- |
| -2.0 kb (-2115~-1852) | Forward | GGGTGTTTACACCTTGTGTG |
|  | Reverse | TGGGGATAATGCTCAGCAGA |
| -1.2 kb (-1306~-1057) | Forward | GCCATACATGAGTTGCAGAG |
|  | Reverse | GGAGTAGAATAGCGATCGTC |
| -0.1 kb (-180~+16) | Forward | TGCACTTGGGTTATGCCAAC |
|  | Reverse | AGGAGTGTTATATGTTGGGC |
