# Supplementary figures and images for: PER1 prevents excessive innate immune response during endotoxin-induced liver injury through regulation of macrophage recruitment in mice
Source: Cell Death Dis. 2016 Apr 7;7(4):e2176–. doi: 10.1038/cddis.2016.9 (PMC4855679; doi:10.1038/cddis.2016.9)

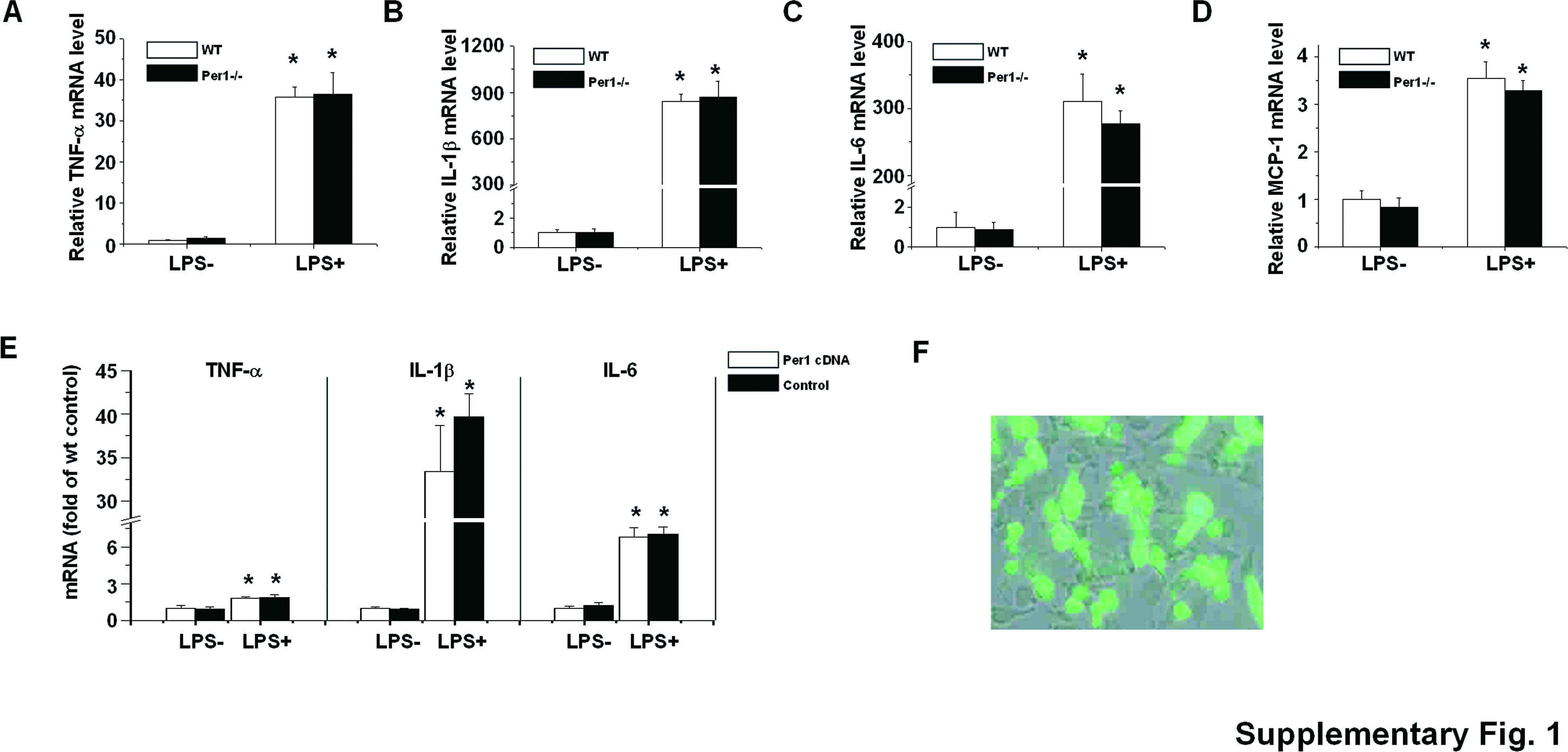

Supplement: Supplementary Figure S1 [file cddis20169x3.tif]

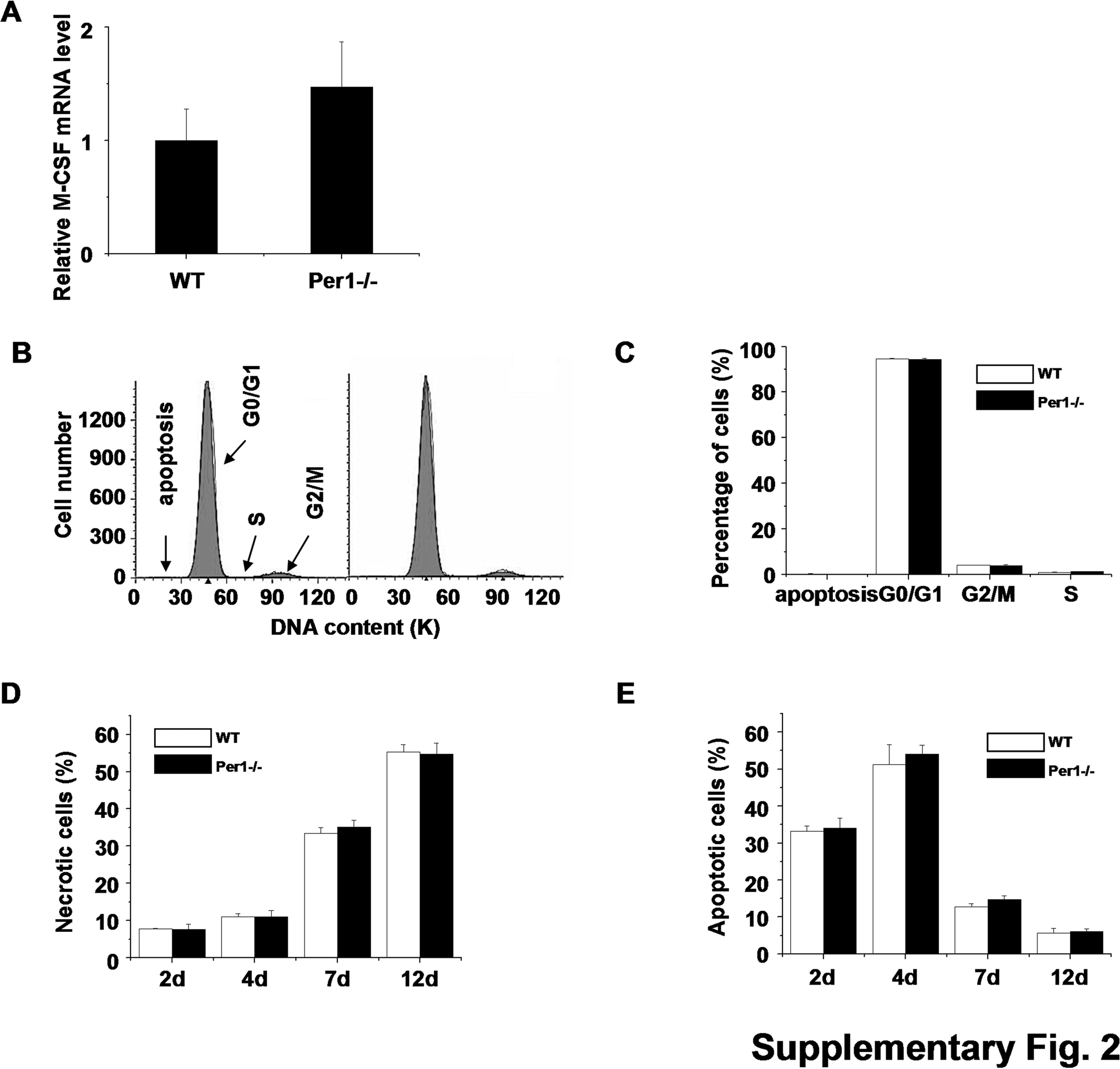

Supplement: Supplementary Figure S2 [file cddis20169x4.tif]
